# Human cap methyltransferase (RNMT) N-terminal non-catalytic domain mediates recruitment to transcription initiation sites

**DOI:** 10.1042/BJ20130378

**Published:** 2013-09-13

**Authors:** Michael Aregger, Victoria H. Cowling

**Affiliations:** Medical Research Council Protein Phosphorylation and Ubiquitylation Unit, College of Life Sciences, University of Dundee, Dow Street, Dundee DD1 5EH, U.K.

**Keywords:** gene expression, methyl cap, transcription, translation, CTD, C-terminal domain, DMEM, Dulbecco's modified Eagle's medium, DRB, 5,6-dichloro-1-β-D-ribofuranosyl benzimidazole, GST, glutathione transferase, HA, haemagglutinin, IMEC, immortalized mammary epithelial cell, HEK, human embryonic kidney, NLS, nuclear localization signal, pol II, polymerase II, RAM, RNMT-activating miniprotein, RNGTT, RNA guanylyltransferase and 5′ phosphatase, RNMT, RNA guanine-7 methyltransferase, TSS, transcriptional start site, WT, wild-type

## Abstract

Gene expression in eukaryotes is dependent on the mRNA methyl cap which mediates mRNA processing and translation initiation. Synthesis of the methyl cap initiates with the addition of 7-methylguanosine to the initiating nucleotide of RNA pol II (polymerase II) transcripts, which occurs predominantly during transcription and in mammals is catalysed by RNGTT (RNA guanylyltransferase and 5′ phosphatase) and RNMT (RNA guanine-7 methyltransferase). RNMT has a methyltransferase domain and an N-terminal domain whose function is unclear; it is conserved in mammals, but not required for cap methyltransferase activity. In the present study we report that the N-terminal domain is necessary and sufficient for RNMT recruitment to transcription initiation sites and that recruitment occurs in a DRB (5,6-dichloro-1-β-D-ribofuranosylbenzimidazole)-dependent manner. The RNMT-activating subunit, RAM (RNMT-activating miniprotein), is also recruited to transcription initiation sites via an interaction with RNMT. The RNMT N-terminal domain is required for transcript expression, translation and cell proliferation.

## Abstract 2

The mRNA methyl cap recruits the mediators of processing events and translation initiation. We report that RNMT, the human cap methyltransferase, is recruited to RNA polymerase II via the N-terminal domain and is required for gene expression and cell proliferation.

## INTRODUCTION

The methyl cap added to the 5′-end of RNA pol II transcripts is required for eukaryotic gene expression. This structure protects mRNA from exonucleases, recruits the protein complexes which mediate RNA-processing events and forms a docking site for the eIF4F (eukaryotic initiation factor 4F) complex to initiate cap-dependent translation [1–3]. The methyl cap is created by the addition of a 7-methylguanosine group to the 5′-end of nascent transcripts, plus methylation of the initial transcribed nucleotides. The 7-methylguanosine group is linked to the first transcribed nucleotide via 5′-to-5′ triphosphate linkage. Transcripts are synthesized with a 5′ triphosphate to which 7-methylguanosine is added in a series of reactions catalysed by two enzymes. RNGTT (RNA guanylyltransferase and 5′ phosphatase) has two enzymic activities: a triphosphatase catalyses removal of the terminal phosphate and a guanylyltransferase catalyses addition of guanosine monophosphate, to create the stucture G(5′)ppp(5′)X (X is the first nucleotide). RNMT (RNA guanine-7 methyltransferase) methylates the guanosine cap on the N-7 position to create the structure m7G(5′)ppp(5′)X [4]. Subsequent methylation of the transcribed nucleotides is catalysed by additional methyltransferases.

Methyl cap formation occurs early during transcription when the phosphorylated RNA pol II CTD (C-terminal domain) recruits RNGTT and RNMT to the nascent transcript as it emerges from the polymerase complex [5–7]. The phosphorylated CTD also activates RNGTT [8]. Although methyl cap formation occurs predominantly at the initiation of transcription, recent evidence has demonstrated that the formation of mRNA cap structures can occur post-transcriptionally [9].

The human cap methyltransferase consists of the catalytic subunit and an obligate activating subunit, RAM (RNMT-activating miniprotein) [10]. The catalytic domain of RNMT, amino acids 121–476, is conserved in sequence, structure and function in all mRNA cap methyltransferases [4]; the loss of viability of cap methyltransferase-deficient *Saccharomyces cerevisiae* can be rescued by expression of the catalytic domain of human RNMT [11]. The RNMT catalytic domain is necessary and sufficient for interaction with its activating subunit RAM [10].

The function of the N-terminal domain of human RNMT, amino acids 1–120, is largely uncharacterized. It is conserved in mammals, but not required for catalytic activity [11]. Cap methyltransferase-deficient *S. cerevisiae* can be rescued by expression of the catalytic domain of human RNMT, with no requirement for the N-terminal domain [11]. Nuclear localization of RNMT is essential for cell viability and the N-terminal domain contains two NLSs (nuclear localization signals). However, these NLS motifs are redundant with a third NLS in the catalytic domain and amino acids 1–120 of RNMT are not required for the nuclear localization of RNMT [12].

In the present study we report that the RNMT N-terminal domain mediates RNMT–RAM recruitment to transcription initiation sites in mammalian cells, and is required for gene expression and cell proliferation.

## MATERIALS AND METHODS

### Cell maintenance

HeLa cells were maintained in DMEM (Dulbecco's modified Eagle's medium) with 10% FBS and HEK (human embryonic kidney)-293 cells in DMEM with 10% FBS and 1 mM sodium pyruvate at 37°C and 5% CO_2_. IMECs (immortalized mammary epithelial cells) were maintained in DMEM/Ham's F12 medium supplemented with 5 μg/ml insulin, 0.5 μg/ml hydrocortisone and 10 ng/ml EGF (epidermal growth factor) [13]. HeLa cells and IMECs were infected with retroviral constructs according to standard protocols, and selected using 0.5 mg/ml G418. HEK-293 cells were transfected using calcium phosphate. Other transfections were carried out with Lipofectamine™ 2000 when cDNA and siRNA co-transfections were performed, RNAiMax when siRNA transfections were performed, and Lipofectamine™ 2000 when cDNA transfections were performed.

### Western blot analysis

Western blots were performed with standard protocols to detect RNMT and RAM (polyclonal anti-sheep antibodies developed in house), monoclonal anti-HA (haemagglutinin) antibodies (Sigma H3663), polyclonal anti-tubulin antibodies (Santa Cruz Biotech, sc-9104), polyclonal anti-c-Myc antibodies (Cell Signaling Technologies, 9402S), polyclonal anti-GST (glutathione transferase) antibodies (anti-sheep antibodies developed in house) and polyclonal anti-(Ser^5^-phosphorylated RNA pol II) antibodies (Chromotek, 3E8).

### Cap methyltransferase assay

The cap methyltransferase assay was performed as described previously [14]. A guanosine-capped, unmethylated substrate ^32^P-labelled on the alpha phosphate (Gp*ppG-RNA) was produced as follows. A total of 200 ng of a 55-base strand of *in vitro*-transcribed RNA was incubated in a 10 μl reaction at 37°C for 30 min with 100 ng of recombinant human RNGTT, 2 μl of (10 μCi) [α-^32^P]GTP and 1 μl of RNAsin (Promega) in reaction buffer [0.05 M Tris/HCl (pH 8.0), 6 mM KCl and 1.25 mM MgCl_2_]. RNA was purified by ammonium acetate precipitation. In the cap methyltransferase assay, 2 ng of capped RNA was incubated with recombinant RNMT or RNMT immunoprecipitated from cell extracts and 100 nM *S*-adenosylmethionine at 37°C for 10 min in reaction buffer. When cell extracts were used, 1×10^6^ HEK-293 cells were transiently transfected by the calcium phosphate method using 6 μg of pcDNA4, pcDNA4 HA–RNMT or pcDNA4 HA–RNMTcat. At 2 days following transfection, cells were lysed and HA–RNMT was immunoprecipitated using anti-HA antibody–agarose conjugate from the quantity of extract indicated in the Figures below.

Following the reaction, RNA was purified, precipitated and resuspended in 4 μl of 50 mM sodium acetate and 0.25 units P1 nuclease for 30 min at 37°C. Cap (Gp*ppG) and methylcap (m7Gp*ppG) were resolved in 0.4 M ammonium sulphate TLC PEI–cellulose (polyethyleneimine–cellulose) plates. Standards were visualized by UV light to establish correct migration. Labelled spots were visualized and quantified by autoradiography, and the percentage conversion of GpppG to m7GppG was calculated. Average results for three independent experiments are shown and error bars indicate the S.D.

### Immunofluorescence

Incubations were performed in 0.25% Tween 20, 0.2% BSA and TBS (Tris-buffered saline) at room temperature (20 °C). IMECs were fixed in 4% paraformaldehyde for 10 min, blocked with 10% donkey serum for 30 min, incubated in 0.16 mg/ml polyclonal sheep anti-HA or anti-RNMT antibodies (developed in house) for 1 h, and incubated with 4 mg/ml Alexa Fluor™ 488-conjugated donkey anti-sheep antibodies (Invitrogen) for 45 min. Cells were counterstained with 1 mg/ml DAPI and visualized by fluorescence microscopy (Zeiss LSM 700).

### ChIP

**Figure 1:**
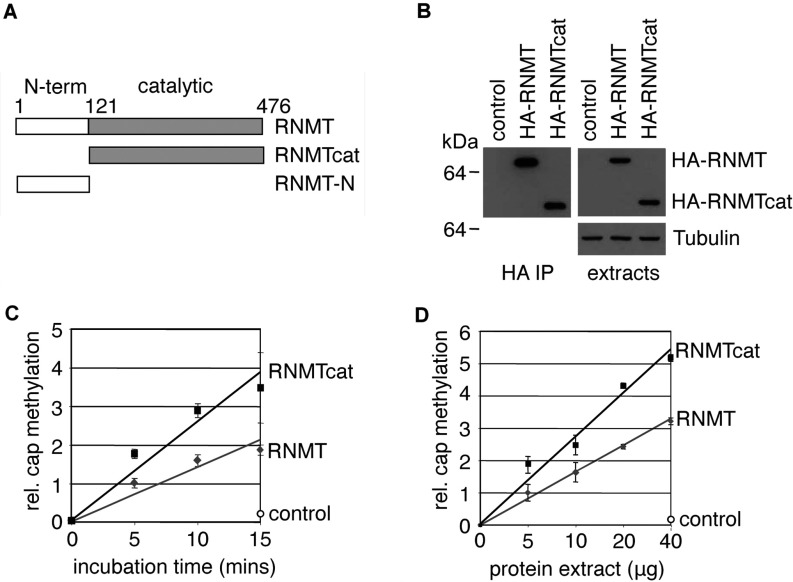
RNMT amino acids 1–120 are not required for catalytic activity (**A**) Human RNMT mutants used in the present study: RNMTcat, amino acids 121–476; and RNMT-N, amino acids 1–120. (**B**) Western blots were performed with anti-HA antibodies to detect expression of HA–RNMT and HA–RNMTcat immunoprecipitated from 50 μg of HEK-293 cell extracts using anti-HA antibodies (HA IP), and in 10 μg cell extracts (extracts). Cap methyltransferase assay was performed on RNMT proteins immunoprecipitated from 10 μg cell extracts or negative control for the time course indicated (**C**) and on RNMT proteins immunoprecipitated from the quantity of cell extracts indicated for 10 min (**D**). The plots depict the means±S.D. for three independent experiments (*P*<0.001 for both plots, Student's *t* test).

HeLa cells (10^6^) were transfected with: (i) 1.5 μg of pcDNA4; (ii) 0.75 μg of pcDNA4 HA–RNMT and 0.75 μg of pcDNA4 FLAG–RAM; (iii) 0.53 μg of pcDNA4 HA–RNMTcat, 0.75 μg of pcDNA4 FLAG–RAM and 0.22 μg of pcDNA4; and (iv) 0.2 μg of pcDNA4 HA–RNMT-N, 0.75 μg of pcDNA4 FLAG–RAM and 0.55 μg of pcDNA4 using Lipofectamine™ 2000 (Invitrogen). Ratios were determined empirically to balance RNMT protein expression (Figures 1B and 3D). ChIPs were performed using the Millipore ChIP kit. anti-HA or anti-FLAG antibody-conjugated agarose (10 μl; Sigma) were utilized to immunoprecipitate the HA–RNMT and FLAG–RAM proteins. DNA was eluted in 50 μl of H_2_O and 2 μl was used per real-time PCR. ChIP signal was determined relative to the input and control immunoprecipitation signal was subtracted. For Figures 3(A) and 3(B), to aid comparison of RNMT WT (wild-type) and RNMTcat, values are expressed relative to RNMT WT for each gene. When used, 50 μM DRB (5,6-dichloro-1-β-D-ribofuranosylbenzimidazole) was incubated with the cells 1 h prior to lysis.

### Real-time PCR

Real-time PCR was performed using Quanta Biosciences SYBR Green FastMix for iQ. All PCR products were sequenced verified. The ChIP primers used were [14]: c-Myc, forward 5′-GCACTGGAACTTACAACACC-3′ (+390) and reverse 5′-ATCCAGCGTCTAAGCAGC-3′ (+531); ruvBL1, forward 5′-TGTGGCCAGTGGACC-3′ (+247) and reverse 5′-ACTTCCCTGAGGAAATAATGG-3′ (+374); cyclinD1, forward 5′-AGCTGCCCAGGAAGAGC-3′ (+186) and reverse 5′-CCGCCTTCAGCATGG-3′ (+312); RNMT, forward 5′-TGAGTGTGACGGCTGGAACTC-3′ (+27) and reverse 5′-CACGCGTTGGGTAGTGAAG-3′ (+164); and c-Myc −2000, forward 5′-AAGACGCTTTGCAGCAAAATC-3′ and reverse 5′-AGGCCTTTGCCGCAAAC-3′.

For real-time PCR analysis of transcripts RNA was extracted using QIAGEN RNeasy mini kit and 500 ng of RNA was converted into cDNA using Quanta qScript cDNA Synthesis kit. cDNA (0.2 μl) was used in real-time PCR reactions. The transcript primers used were: c-Myc, forward 5′-TCTGAGGAGGAACAAGAA-3′ and reverse 5′-GAAGGTGATCCAGACTCT-3′; Top2a, forward 5′-GATGCAGGGGGCCGAAACTCC-3′ and reverse 5′-CCCAACCACACCAAGGCCTGAA-3′; Troap, 5′-AGGGGCCTCGGTAAGCCATCA-3′ and reverse 5′-CAGTGGGGAAAGGCGTGCGT-3′, Rad21, forward 5′-CAGCTATGCCTCCACCACCACC-3′ and reverse 5′-GCTGAGGAGGCATCACAGGCTCT-3′; RuvBL1, forward 5′-CATTGGGCTGCGAATAAAG-3′ and reverse 5′-TCTGTCTCACACGGAGTT-3′; Gapdh (glyceraldehyde-3-phosphate dehydrogenase), forward 5′-GGAGTCAACGGATTTGG-3′ and reverse 5′-GTAGTTGAGGTCAATGAAGGG-3′.

### [^35^S]methionine incorporation

Cells (1×10^5^) were transfected with 1 μg of cDNA in the ratios described above. [^35^S]methionine (11 μCi; Perkin Elmer) were incubated with the cells for 15 min. Protein was harvested and counts incorporated in macromolecules were determined.

### Glutathione–Sepharose pull down

HeLa cells were lysed in hypotonic lysis buffer [20 mM Hepes (pH 7.5), 10 mM KCl, 1.5 mM MgCl_2_, 1 mM EDTA and 1 mM EGTA] and nuclear proteins were extracted in Triton X-100 lysis buffer [10 mM Tris (pH 7.05), 50 mM NaCl, 30 mM sodium pyrophopshate, 50 mM NaF, 10% glycerol, 0.5% Triton X-100, 10 μM leupeptin and 1 μM pepstatin and aprotinin, supplemented with Phosphatase Inhibitor Cocktail II and III; Sigma]. Precleared nuclear extract (2 mg) was incubated with glutathione–Sepharose 4B (GE Healthcare) and purified recombinant GST, GST–RNMT or GST–RNGTT. Recombinant protein was produced as described in [10].

### Transformation assays

Transformation assays were performed as described in [14].

### Transfections for cell counts

HeLa cells (5×10^4^) were plated in six-well plates and the following day transfected with 200 pmol of RAM siRNA (Dharmacon) and 1 μg of DNA using Lipofectamine™ 2000. IMECs (5×10^4^) were plated in six-well plates and transfected the following day with 200 pmol of RNMT siRNA (Dharmacon) using RNAimax (Invitrogen). Both transfections were performed according to the manufacturer's instructions. RAM and RNMT cDNAs were used which were resistant to siRNAs due to mutation of the wobble codon (which left the amino acid sequence intact). To balance RNMT expression, the following transfection ratios were used; 1 pcDNA4 HA–RNMT:1 pcDNA4 FLAG–RAM; and 0.7 pcDNA4 HA–RNMTcat:1 pcDNA4 FLAG–RAM:0.3 pcDNA4. Cells were counted using a haemocytometer and a Countess cell counter (Invitrogen).

## RESULTS

The human cap methyltransferase complex, RNMT–RAM, is required for gene expression and mammalian cell proliferation [10,12]. Human RNMT has a defined cap methyltransferase domain (amino acids 121–476), but the function of the RNMT N-terminal domain (amino acids 1–120) is unclear. In the following study, the function of the RNMT N-terminus was characterized in human cell lines by comparing the action of full-length human RNMT (amino acids 1–476), with the minimal catalytic domain (amino acids 121–476) and the N-terminal domain RNMT-N (amino acids 1–120) (Figure 1A).

### RNMT N-terminal domain is not required for catalytic activity

We investigated whether the RNMT N-terminal domain influences cap methyltransferase activity. In a previous publication, removing the N-terminal domain of RNMT reduced the activity of recombinant RNMT 2-fold; however, its activity in mammalian cells had not been reported [11]. Enzymes expressed in mammalian cells can have differential activity to those expressed in bacteria for a variety of reasons including the effects of mammalian-specific post-translational modifications and correct protein folding. The influence of the RNMT N-terminal domain on cap methyltransferase activity in human cell extracts was investigated by comparing the activity of full length human RNMT with the N-terminal deletion mutant RNMTcat (Figure 1A). HEK-293 cells were transfected with HA-tagged RNMT cDNAs. Following transfection, RNMT proteins were judged to be equivalently expressed by Western blot detection of the HA tag in cell extracts (Figure 1B). Immunoprecipitation of RNMT and RNMTcat using anti-HA antibodies also produced equivalent levels of RNMT protein (Figure 1B). The enzymic activity of immunoprecipitated HA–RNMT and HA–RNMTcat was compared in an *in vitro* cap methyltransferase assay. The methylation of a capped RNA substrate was measured over a time course (Figure 1C) and over a titration of immunoprecipitated HA–RNMT protein (Figure 1D). In agreement with data published using recombinant RNMT [11], HA–RNMTcat retained cap methyltransferase activity (Figures 1C and 1D). Control immunoprecipitations did not exhibit significant cap methyltransferase activity. In contrast with measurements made with recombinant proteins in which RNMTcat exhibited a 2-fold reduction in cap methyltransferase activity compared with RNMT WT [11], cellular RNMTcat exhibited a 1.5–2-fold increase in cap methyltransferase activity compared with the WT protein. This difference may be owing to differences in protein folding or post-translational modifications.

**Figure 2:**
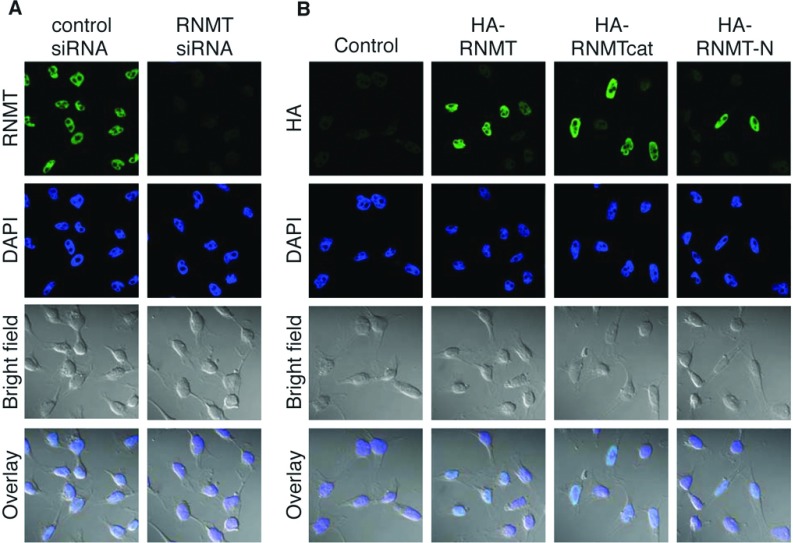
RNMT nuclear localization is mediated independently by the N-terminal and catalytic domains (**A**) Immunofluoresence analysis was performed on HeLa cells transfected with control or RNMT-directed siRNA for 48 h. The subcellular localization of RNMT was detected with anti-RNMT antibodies. (**B**) Immunofluoresence analysis was performed on HeLa cells transfected with pcDNA4 (control) or pcDNA4 HA–RNMT, HA–RNMTcat and HA–RNMT-N plus pcDNA4 FLAG–RAM. The subcellular localization of RNMT was detected via the HA tag. In (**A**) and (**B**) DAPI stain was used to detect nuclei and a bright field view to detect the cell membrane.

RNMT is required to be nuclear to function and has three putative NLSs, two of which are present in the N-terminal 120 amino acids [12]. The subcellular localization of endogenous RNMT was investigated using immunofluorescence. As expected, RNMT was found predominantly in the nucleus (Figure 2A). Detection of RNMT was verified by loss of signal in RNMT-depleted cells. The subcellular localization of HA-tagged RNMT, RNMTcat and RNMT-N was compared by immunofluorescence performed to detect the HA tag. HA–RNMT, HA–RNMTcat and HA–RNMT-N were all observed to be nuclear (Figure 2B).

### RNMT N-terminus is required for recruitment to chromatin

**Figure 3:**
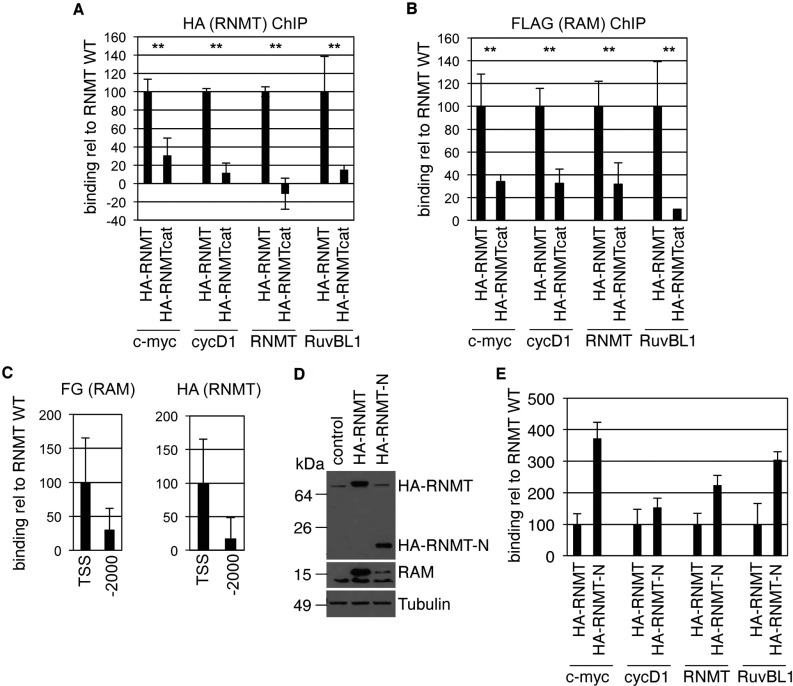
RNMT is recruited to chromatin via the N-terminus ChIP was performed on HeLa cells transfected with pcDNA4 HA–RNMT, HA–RNMTcat or pcDNA4 (control), plus pcDNA4 FLAG–RAM. Immunoprecipitations were performed with antibodies raised against HA to detect RNMT (**A**) and FLAG to detect RAM (**B**). PCR was performed against the promoter-proximal region of the genes indicated. PCR signal relative to input, subtracted from negative control immunoprecipitation is depicted as means±S.D. for three independent experiments (**P<0.01, Student's *t* test). (**C**) For the same immunoprecipitations, binding to the c-Myc gene TSS and 2000 bases upstream of the start site (−2000) was compared. Results are an average of three independent experiments and the error bars indicate S.D. (**D**) HeLa cells were transfected with pcDNA4 HA–RNMT, HA–RNMT-N or vector control plus pcDNA4 FLAG–RAM and Western blotting was performed to detect RNMT proteins, RAM and tubulin. Molecular masses are shown on the left-hand side in kDa. (**E**) ChIPs were performed using anti-HA antibodies as above. Results are an average of three independent experiments and the error bars indicate S.D.

RNMT has been observed to interact with RNA pol II and RNGTT, and is recruited to transcription initiation sites where it methylates the nascent transcript [15–18]. These experiments were performed before the discovery that RNMT is only isolated in a complex with its activating subunit RAM [10]. In order to investigate whether the RNMT N-terminus influences RNMT–RAM recruitment to transcription initiation sites, HeLa cells were transfected with HA–RNMT or HA–RNMTcat and ChIP was performed using anti-HA antibodies to detect chromatin-recruited RNMT (Figure 3A). To provide independent confirmation of the RNMT–RAM complex, FLAG–RAM was co-transfected with RNMT cDNAs and ChIP was performed with anti-FLAG antibodies (Figure 3B). HA–RNMT and HA–RNMTcat were expressed equivalently, as was FLAG–RAM, in these transfections (Figure 6A, see the anti-HA blot since anti-RNMT antibodies predominantly recognize the N-terminal domain). Recruitment of RNMT and RAM to the transcription initiation sites of four genes chosen as random was investigated; c-Myc, cyclin D1, RuvBL1 and RNMT. ChIP values were expressed as binding relative to the RNMT WT transfection. HA–RNMT and FLAG–RAM were recruited to the transcription initiation sites of all four genes (Figures 3A and 3B). RNMTcat–RAM recruitment to transcription initiation sites was strongly impaired, despite being expressed equivalently to the WT complex (Figure 6A), and therefore the N-terminus of RNMT is required for effective RNMT–RAM recruitment to chromatin (Figures 3A and 3B). This result also demonstrates that RAM cannot be recruited to transcription initiation sites independently of RNMT. As a negative control, RNMT and RAM recruitment to the c-Myc TSS (transcriptional start site) and 2000 bases upstream of the c-Myc transcription initiation site (−2000) was compared (Figure 3C). c-Myc −2000 is a location which had previously been demonstrated to have reduced RNMT–RAM binding [15]. HA–RNMT and FLAG–RAM were not significantly recruited to this site (Figure 3C).

The most likely explanation for reduced RNMTcat recruitment to transcription initiation sites is that the N-terminal domain is mediating recruitment. In order to test this hypothesis HeLa cells were transfected with HA-tagged RNMT N-terminal domain, HA–RNMT-N (amino acids 1–120), resulting in expression equivalent to HA–RNMT (Figure 3D). RNMT stabilizes RAM and, consistent with HA–RNMT but not HA–RNMT-N interacting with RAM, only expression of HA–RNMT increases RAM expression [10] (Figure 3D). Using ChIP performed using anti-HA antibodies, HA–RNMT-N was observed to be recruited to transcription initiation sites at an equivalent level to HA–RNMT (Figure 3E). This experiment further confirms that RAM is not required for RNMT recruitment to chromatin since RNMT-N does not interact with RAM.

**Figure 4:**
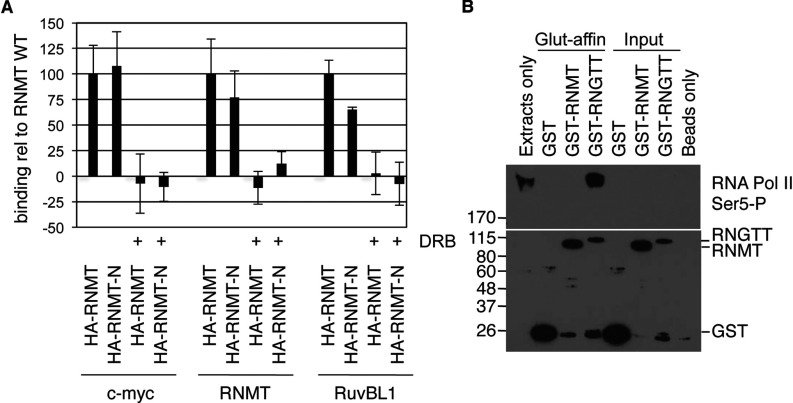
RNMT recruitment to chromatin is inhibited by DRB (**A**) ChIPs were performed on HeLa cells transfected with pcDNA4 HA–RNMT, HA–RNMT-N or pcDNA4 (control), plus pcDNA4 FLAG–RAM. Cells were treated with 50 μM DRB or vehicle control for 1 h prior to lysis. Immunoprecipitations were performed with anti-HA antibodies. PCR signal relative to input, subtracted from negative control immunoprecipitation is depicted as means±S.D. for three independent experiments (comparison with and without DRB resulted in *P* values<0.05 for the HA–RNMT and HA–RNMT-N transfections, Student's *t* test). (**B**) Purified recombinant GST, GST–RNMT and GST–RNGTT were incubated with HeLa nuclear cell extracts and glutathione pull downs were performed. Western blots were performed to detected RNA pol II Ser^5^ CTD phosphorylation and GST. Molecular masses are shown on the left-hand side in kDa.

*In vitro*, RNMT has been demonstrated to be recruited to phosphorylated transcribing RNA pol II [17,18]. In order to assess the effect of RNA pol II phosphorylation on RNMT recruitment, cells were treated for 1 h with 50 μM DRB, which significantly inhibits RNA pol II CTD phosphorylation [19]. HA–RNMT and HA–RNMT-N recruitment to transcription initiation sites was significantly reduced by this treatment, suggesting that RNMT recruitment to the sites which we investigated is dependent on RNA pol II phosphorylation (Figure 4A). A direct interaction of mammalian RNMT and RNA pol II has not been reported to date, although a previous publication reported RNMT and RNA pol II co-immunoprecipitation with the human mRNA cap guanylyltransferase RNGTT (see Figure 4 in [16]). This suggests that RNMT is either recruited to RNA pol II via RNGTT or is in a complex with both proteins. We attempted to discern whether RNMTcat is defective for interaction with RNGTT and/or RNA pol II. Using recombinant GST–RNGTT as bait we were able to detect a robust interaction with Ser^5^ CTD-phosphorylated RNA pol II (Figure 4B). However, we were not able to detect an interaction between recombinant GST–RNMT and RNA pol II (Figure 4B). Despite extensive efforts, we were also unable to detect an interaction between recombinant RNMT and RNGTT, either in the presence or absence of RNA pol II, nor were we able to detect an interaction between cellular RNMT and RNA pol II in mammalian cell extracts, whether endogenous or overexpressed (results not shown). Our and others’ previous research has clearly demonstrated that RNMT is recruited to transcription initiation sites [15], and that cap methylation in mammals occurs co-transcriptionally [17,18,20]. However, the precise molecular details of how RNMT is recruited to transcription initiation sites remains unclear.

### The RNMT N-terminus is required for transcript expression and mRNA translation

**Figure 5:**
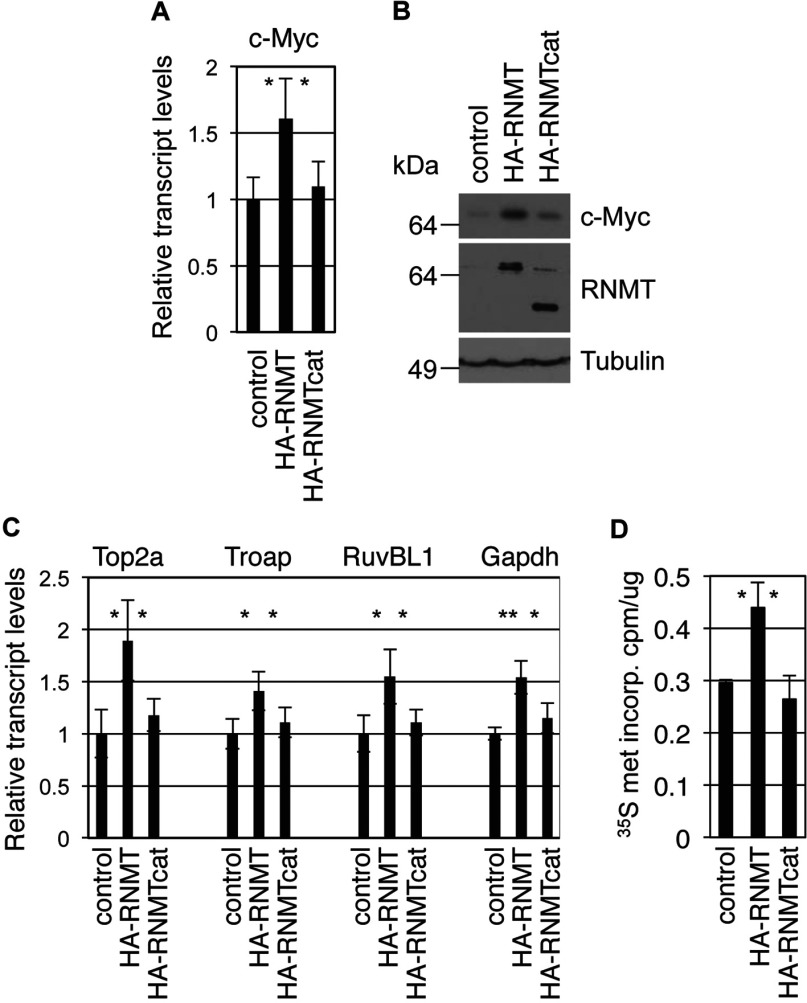
The RNMT N-terminus is required for gene expression HeLa cells were transfected with pcDNA4 HA–RNMT, pcDNA4 HA–RNMTcat and vector control plus pcDNA4 FLAG–RAM WBL (siRNA-resistant DNA). (**A**) At 24 h after transfection, RNA was harvested and real-time PCR was performed to detect expression of the c-Myc transcripts. Results are an average of three independent experiments and the error bars indicate the S.D. (**B**) Western blots were performed to detect c-Myc, RNMT and tubulin. Molecular masses are shown on the left-hand side in kDa. (**C**) As in (**A**) except performed with the genes indicated. (**D**) [^35^S]Methionine *in vivo* labelling was performed to estimate amino acid incorporation rates in the same experimental system. Results are means±S.D. for three independent experiments (**C**) and a representative of three independent experiments performed in triplicate (**D**). *P<0.05 and **P<0.01 when comparing HA–RNMT with the vector control or HA-RNMTcat, Student's *t* test.

RNMT and the methyl cap increase transcript abundance by promoting transcription and by stabilizing transcripts [21,22]. Methyl cap formation occurs co-transcriptionally and since the RNMT N-terminus mediates chromatin recruitment to transcription initiation sites, RNMTcat-expressing cells should be defective for transcript expression. Initially expression of c-Myc was investigated, since this is rate-limiting for cell proliferation, and has been demonstrated previously to be responsive to RNMT expression [10]. Cells were transfected with HA–RNMT and RAM or HA–RNMTcat and RAM, RNA was harvested and real-time PCR was performed. The c-Myc transcript was found to be up-regulated in response to HA–RNMT, but not HA–RNMTcat (Figure 5A). Consistent with the transcript data, c-Myc protein was also found to be up-regulated in response to HA–RNMT, but not HA–RNMTcat (Figure 5B). For five more genes chosen at random, expression of HA–RNMT increased transcript expression significantly, whereas HA–RNMTcat expression did not (Figure 5C). Consistent with RNMTcat being defective for promoting transcription and c-Myc protein expression, this mutant was also defective for promoting net amino acid incorporation into macromolecules (Figure 5D).

### RNMT N-terminus is required for cell proliferation

**Figure 6:**
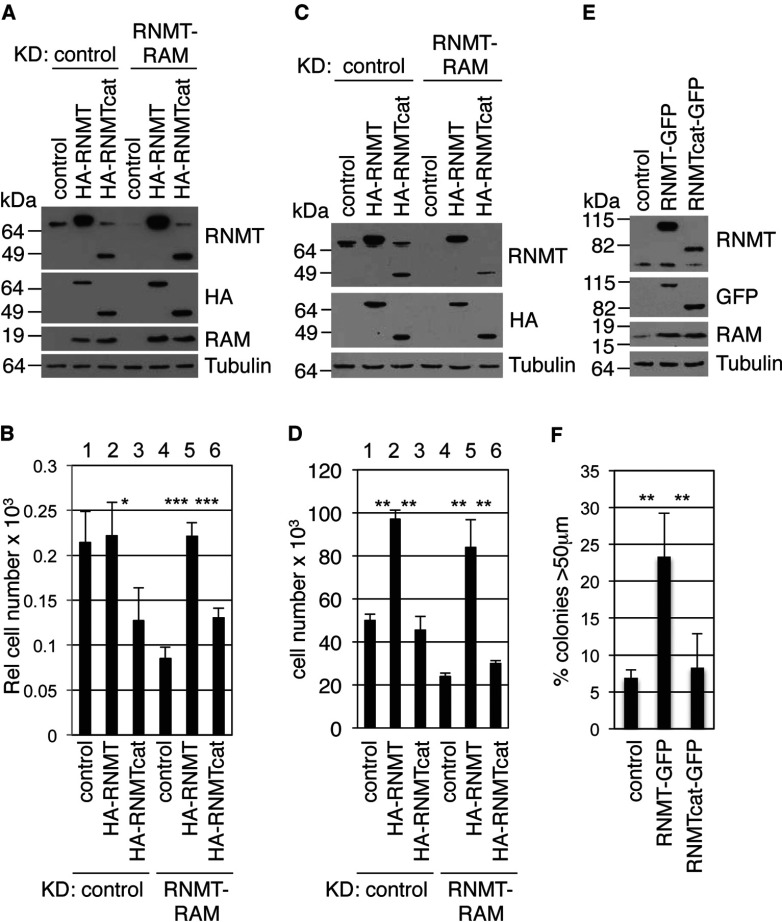
RNMT amino acids 1–120 are required for cell proliferation RNMT–RAM was knocked down in HeLa cells using RAM-directed siRNA or a control, and cells were transfected with pcDNA4 HA–RNMT, pcDNA4 HA–RNMTcat or a vector control, and pcDNA4 FLAG–RAM WBL (siRNA-resistant cDNA). After 3 days Western blots were performed to detect the antigens indicated (**A**) and cells were counted (**B**). RNMT–RAM was knocked down using RNMT-directed siRNA in IMEC expressing HA–RNMT and HA–RNMTcat and control lines. After 3 days Western blots were performed to detect the antigens indicated (**C**) and cells were counted (**D**). (**E**) IMECs (5×10^3^) expressing RNMT–GFP and RNMTcat–GFP or control lines were plated in soft agar. (**F**) At 10 days later the number of established colonies (over 50 μm) were counted. Histograms depict mean±S.D. for at least three independent experiments. **P<0.01 and ***P<0.001, Student's *t* test. Molecular masses are shown on the left-hand side of the blots in kDa.

Since RNMTcat is defective for supporting gene expression we investigated whether it is required for supporting cell proliferation. In HeLa cells, endogenous RNMT–RAM expression was reduced by transfection of siRNA directed against RAM. Expression of RNMT and RAM are co-dependent, thus inhibiting expression of one component of the complex results in loss of both [10] (Figure 6A). Endogenous RNMT–RAM was replaced with exogenously expressed HA–RNMTcat and RAM, or HA–RNMT and RAM. Expression of HA–RNMT and HA–RNMTcat was judged to be equivalent by Western blotting performed using anti-HA antibodies (Figure 6A, note that the anti-RNMT antibodies predominantly recognize the RNMT N-terminal domain and therefore have reduced affinity for RNMTcat compared with RNMT WT. The anti-HA antibody Western blots should be inspected to judge the relative expression of HA–RNMT and HA–RNMTcat). Two days following transfection, cells were counted; transfection of HA-RNMT in the presence of endogenous RNMT had no effect on cell proliferation, whereas transfection of HA–RNMTcat inhibited cell proliferation (Figure 6B, lanes 1–3). A reduction in RNMT expression resulted in a reduction in cell proliferation and this was rescued by expression of HA–RNMT, but not HA–RNMTcat (Figure 6B, lanes 4–6).

The ability of RNMTcat to support cell proliferation was also investigated in a non-transformed mammary epithelial cell line (IMECs) [13]. In IMECs, endogenous RNMT–RAM was knocked down using RNMT-directed siRNA in lines expressing siRNA-resistant HA–RNMT and HA–RNMTcat (Figure 6C). Three days following transfection cells were counted; HA–RNMT expression resulted in an increase in cell proliferation, whereas HA–RNMTcat did not (Figure 6D, lanes 1–3). As in HeLa cells, knock down of RNMT–RAM resulted in a loss in cell proliferation which was rescued by expression of HA–RNMT, but not HA–RNMTcat (Figure 6D, lanes 4–6). In agreement with previous data, RNMTcat–RAM did not induce apoptosis in HeLa cells or IMECs (results not shown, [12]). A key difference exhibited by IMECs was that in the absence of RNMT–RAM knock down, exogenous expression of HA–RNMT increases the proliferation rate. The reasons for this are not yet clear, although obviously reflect a difference in dependence on RNMT for cell proliferation.

IMECs are a non-transformed epithelial line and cannot proliferate in suspension. We previously demonstrated that exogenous RNMT expression permits anchorage-independent proliferation and wanted to determine whether this is dependent on the N-terminal domain [14]. RNMT–GFP is used in preference to HA–RNMT in anchorage-independent proliferation assays since it results in more robust transformation than HA–RNMT, probably due to enhanced expression [14]. RNMT–GFP and RNMTcat–GFP were expressed in IMECs, with the result that RNMT–GFP permitted anchorage-independent cell proliferation, whereas RNMTcat–GFP did not (Figures 6E and 6F). Collectively, these data demonstrate that the N-terminal domain of RNMT is required for supporting cell proliferation, consistent with its requirement for recruitment to chromatin and gene expression.

## DISCUSSION

mRNA methyl cap synthesis was defined biochemically in 1970s, however, the basics of the cellular mechanism are still being uncovered [23]. Formation of the mRNA methyl cap occurs predominantly whilst transcripts are being synthesized, when RNGTT and RNMT are recruited to the transcribing RNA pol II [15,17,18]. The mechanism by which these enzymes are recruited to transcription initiation sites is arguably as important as the enzyme activity for cellular gene expression. In the present study we report that the non-catalytic N-terminal domain of RNMT is required for efficient recruitment of the cap methyltransferase complex, RNMT–RAM, to transcription initiation sites. Removal of the N-terminal domain results in reduced recruitment of RNMT to transcription initiation sites, whereas the N-terminal domain expressed alone is recruited equivalently to the WT protein. RNMT is only found in a complex with RAM and, perhaps unsurprisingly, we observed RAM recruitment to transcription initiation sites. However, RAM recruitment is dependent on RNMT since it is only recruited significantly when co-expressed with RNMT WT and not when expressed with RNMTcat. Conversely, RNMT recruitment does not require RAM since the RNMT N-terminal domain is recruited, but does not bind to RAM.

The catalytic domains of mammalian *S. cervisiae* and *Schizosaccharomyces pombe* cap methyltransferases are highly homologous in sequence and function [4,11]. Conversely their N-terminal domains are not well conserved. The question then arises of how the *S. cerevisiae* and *S. pombe* cap methyltransferases are recruited to transcription initiation sites. Recruitment of *S. cerevisiae* and *S. pombe* cap methyltransferases is dependent on RNA pol II phosphorylation [24,25], and in the present study we demonstrate that this is also the case for RNMT (Figure 4A), which suggests that recruitment of cap methyltransferases shares some common mechanism. We highlight that the catalytic domain of RNMT, RNMTcat, is still recruited to promoters, but with significantly reduced efficiency compared with the WT protein. In *S. cerevisiae and S. pombe*, the catalytic domain alone may be sufficient for effective cap methyltransferase recruitment to transcription initiation sites. The complexity of the transcriptional apparatus increased significantly with vertebrate evolution and we speculate that the N-terminal domain of RNMT may have evolved to maintain cap methyltransferase recruitment to transcription initiation sites when challenged with the mammalian RNA pol II machinery. There are other examples of significant differences between the capping enzymes in different species. For example, the *S. pombe* cap methyltransferase is found in a complex with the elongation factor P-TEFb, whereas analogous interactions have not been observed in *S. cerevisiae* or mammalian cells [25].

An obvious extension to the present study to clarify the role of the RNMT N-terminal domain would be to investigate its influence on the interaction between RNMT, RNA pol II and RNGTT. *In vitro*, RNMT is recruited to the phosphorylated, elongating RNA pol II [17,18]. However, despite extensive efforts, we were unable to detect a direct interaction of recombinant or cellular RNMT with RNA pol II and/or RNGTT, although an interaction between RNGTT and RNA pol II was readily observed (Figure 4B). Although an interaction between the *S. cerevisiae* cap methyltransferase (ABD1) and RNA pol II has been observed [26], only a weak interaction between mammalian RNMT, RNGTT and RNA pol II has been reported [16]. This suggests that there may be as yet unidentified factors recruiting RNMT to the transcribing polymerase, or that this interaction may require a chromatin context. Indeed, in the present study a relationship between RNMT and phosphorylated RNA pol II is observed in cells; treatment with the RNA pol II kinase inhibitor DRB reduced RNMT recruitment to promoters (Figure 4A). However, the relationship between RNMT and RNA pol II is complex; in a prior study, treatment with DRB for 8 h did not inhibit recruitment of RNMT [15]. This may be a consequence of increased promoter pausing of RNA pol II in addition to inhibition of phosphorylation.

The predominant finding of the present study is that the RNMT N-terminal domain is required for the recruitment of RNMT–RAM to transcription initiation sites. We also observed that the N-terminal domain negatively regulates cap methyltransferase activity. An intriguing possibility is that cellular signalling pathways use this domain to influence mRNA cap methylation.
